# Current understanding of adult neurogenesis in the mammalian brain: how does adult neurogenesis decrease with age?

**DOI:** 10.1186/s41232-020-00122-x

**Published:** 2020-06-18

**Authors:** Yoshitaka Kase, Takuya Shimazaki, Hideyuki Okano

**Affiliations:** 1 Department of Physiology, Keio University School of Medicine, 35 Shinanomachi, Shinjuku-ku, Tokyo, 160-8582 Japan; 2 Department of Geriatric Medicine, Graduate School of Medicine, The University of Tokyo, Bunkyo-ku, Tokyo, 113-8655 Japan

**Keywords:** Adult neurogenesis, Aging, Neural stem cell, Subgranular zone, Transit amplifying progenitor cell, Ventricular-subventricular zone

## Abstract

Adult neurogenesis occurs throughout life in restricted brain regions in mammals. However, the number of neural stem cells (NSCs) that generate new neurons steadily decreases with age, resulting in a decrease in neurogenesis. Transplantation of mesenchymal cells or cultured NSCs has been studied as a promising treatment in models of several brain injuries including cerebral infarction and cerebral contusion. Considering the problems of host-versus-graft reactions and the tumorigenicity of transplanted cells, the mobilization of endogenous adult NSCs should be more feasible for the treatment of these brain injuries. However, the number of adult NSCs in the adult brain is limited, and their mitotic potential is low. Here, we outline what we know to date about why the number of NSCs and adult neurogenesis decrease with age. We also discuss issues applicable to regenerative medicine.

## Background

In mammals, neural stem cells (NSCs) in the early embryonic period are called neuroepithelial cells. Neuroepithelial cells self-renew symmetrically on the ventricular surface [1, 2]. This symmetric division increases the number of neuroepithelial cells lining the ventricular surface and enlarges the ventricular zone (VZ) [3]. After the neural tube is closed, neuroepithelial cells are converted into radial glial cells with long radial fibers, and asymmetric cell division that allows the generation of a large number of neurons also begins [4]. After the neurogenic period, these radial glial cells differentiate into glia, astrocytes with NSC properties, or ependymal cells with cilia [5].

In the adult brain, most NSCs are quiescent. However, NSCs in the ventricular-subventricular zone (V-SVZ) and subgranular zone (SGZ) of the hippocampal dentate gyrus (DG) slowly divide to generate transit amplifying progenitor cells (TAPs) via a state called activated neural stem cells (aNSC) and thus generate new neurons [6–8]. Such adult neurogenesis in the mammalian brain was first suggested in the 1960s [9], and neurogenesis has been found to occur primarily in the V-SVZ and SGZ throughout life [10–15]. New neurons generated in these two neurogenic areas are incorporated into neural circuits and play important roles. For example, in rodents, new neurons born in the V-SVZ migrate into the olfactory bulb via the rostral migratory stream (RMS), in which newly generated neurons migrate along each other as oriented chains and are encapsulated by a complex network of astrocyte tunnels [14, 16]. The attenuation of adult neurogenesis in the V-SVZ has been reported to cause abnormal olfactory and sexual behavior in mice [17]. In addition, new neurons generated in the SGZ are also integrated into DG neural circuits and play an important role in the formation of short-term memory. The attenuation of neurogenesis in the mouse SGZ has been reported to result in the impairment of new memory formation [7, 18–23]. These new neurons are also important for the formation of spatial memories [24–27]. Furthermore, new integrated neurons in the DG have the function of organizing past memories and alleviating the stress response [28, 29]. However, adult neurogenesis decreases with age, mainly due to a decrease in NSCs and TAPs [14, 30, 31]. Several studies have reported that this reduction is likely to be caused by decreases in extrinsic signals that support the proliferation of NSCs, including mitotic signals such as EGF and FGF-2 [32, 33], and increases in systemic pro-aging factors [34].

Here, we review adult NSCs and neurogenesis and the mechanisms of their age-related declines. We will also describe the challenges of NSC activation as a therapeutic strategy.

## Main text

### Origin of adult NSCs

NSCs are actively self-renewing, allowing the generation of a large number of neurons and glia during central nervous system development [35] and thus rapid cerebral development during the embryonic stage. Although the growth of the brain continues even after birth, it slows quickly even in the V-SVZ and SGZ and is completed by approximately 4 weeks after birth in mice. After this developmental phase, active neurogenesis via TAPs generated from slowly dividing NSCs occurs only in the V-SVZ and SGZ [14, 22, 30, 31, 36–38]. It was previously believed that these slowly dividing adult NSCs merely remain actively dividing embryonic NSCs [39]. However, it was recently reported that slowly dividing embryonic NSCs with high p57 expression become dormant adult NSCs in the V-SVZ [40]. In addition, it has been suggested that the cleavage plane orientation of embryonic radial glial cells regulates the number of adult NSCs in the lateral ganglionic eminence [41]. In the mouse SGZ, NSCs originate from Sonic Hedgehog-responsive progenitors expressing Gli1 located in the ventral hippocampus during late gestation [42].

### Age-dependent decrease in neural stem cells and adult neurogenesis

Although the biological significance of adult hippocampal neurogenesis in humans is still under debate [43, 44], the physiological roles of adult neurogenesis in other species are evident.

On the other hand, the number of NSCs decreases with age in both the V-SVZ [14, 22, 30, 31, 36–38] and SGZ [38, 45, 46], resulting in a reduction in neurogenesis. What factors contribute to the decrease in adult neurogenesis upon aging? The expression of EGF and FGF, which are well-known mitogens that promote the self-renewal of aNSCs and TAPs, in the brain decreases with age, which may be a cause of the age-dependent decline in neurogenesis [32, 33, 42].

It has also been reported that the sharing of blood circulation between old and young mice (parabiosis) improves brain function and other functions in old mice [47]. The circulation of blood from young mice through the cardiovascular system of aged mice promotes neurogenesis in the SGZ and activates neural functions. In this study, C-C motif chemokine ligand 11 (CCL11) was reported to be an aging-promoting factor. β2-microglobulin has also been identified as a pro-aging factor that promotes age-dependent declines in neurogenesis in the SGZ and cognitive function [48]. In contrast, another study using parabiosis identified GDF11 (a circulating TGF-β family member) as an anti-aging factor that can improve the cerebral vasculature and enhance neurogenesis in the V-SVZ of aged mice [49]. More recently, Yousef et al. showed that an age-dependent increase in the soluble form of vascular cell adhesion molecule 1 (VCAM1), which is a protein that promotes interaction between blood vessels and immune cells in plasma, may cause an age-related decrease in hippocampal neurogenesis via an increase in the inflammatory transcriptional profile, including the transcription of VCAM1, in endothelial cells in the mouse hippocampus [50]. The age-related decrease in adult neurogenesis caused by changes in the components of plasma is likely to be mediated at least in part by changes in DNA methylation status. It has been shown that an age-dependent decrease in the expression of ten-eleven translocation methylcytosine dioxygenase 2 (Tet2), which catalyzes the production of 5-hydroxymethylcytosine, is one of the causes of the age-related decline in neurogenesis in the mouse SGZ [51]. Interestingly, in this study, heterochronic parabiosis restored Tet2 expression and neurogenesis in the aged hippocampus.

Adult NSCs in the V-SVG and SGZ reside in specialized microenvironments referred to as stem cell niches, which are essential for the maintenance of NSCs and the control of neurogenesis. A number of studies have suggested that blood vessels are an integral part of stem cell niches. For instance, the vascular endothelial growth factor VEGF is known to be a glycoprotein that promotes angiogenesis and has a positive effect on neurogenesis [52–54].

In addition, cerebral blood volume in the DG has been shown to be correlated with neurogenesis in the SGZ [55]. Thus, vascular aging may slow the growth of NSCs and TAPs and diminish their ability to generate new neurons. Indeed, it has been shown that the VEGF level in the hippocampus decreases upon aging in rats [56].

Proteostasis has also been shown to be important for the regulation of NSC proliferation and differentiation. In the adult brain, NSCs in a relatively quiescent state need to become aNSCs and self-renew or divide into TAPs to generate new neurons. Leeman et al. showed that aNSCs have active proteasomes but that quiescent NSCs (qNSCs) accumulate protein aggregates, many of which are stored in large lysosomes. Upon aging, qNSCs display lysosomal defects, increased accumulation of protein aggregates, and decreased ability to be activated. It has been reported that enhancing the lysosomal pathway in aged qNSCs improves their ability to be activated [57].

Mitochondrial dysfunction may also be a cause of the age-related decline in adult neurogenesis. In the V-SVZ, mitochondrial abundance and the oxygen consumption rate decrease with age, but the mutation rate of mitochondrial DNA in aged neural progenitor cells does not increase [58]. It has also been suggested that mitochondrial dysfunction in the SGZ induced by aging may be responsible for decreased neurogenesis [59].

Oxygen levels, which affect oxidative stress via the production of reactive oxygen species in stem cell niches, alter the fate of NSCs. HIF-1α responds to hypoxic conditions and promotes NSC proliferation. HIF-1α regulates adult neurogenesis by promoting NSC/progenitor cell proliferation and differentiation via Wnt/β-catenin signaling [60]. Another study revealed that NSC-encoded HIF-1α is essential for the maintenance of adult neurogenesis in the V-SVZ and demonstrated that NSCs within the SVZ maintain the integrity of their vascular niche via HIF-1α signaling [61].

There have also been studies on sex hormones. While androgen, a male hormone, has been shown to positively regulate neurogenesis in the SGZ of male mice, circulating androgen levels decrease with age. Interestingly, the administration of androgen increases adult neurogenesis only in young male rats but not in female or aged male rats, even though androgen receptor expression increases in the hippocampus of both male and female rats after middle age. Aging may result in a loss of reactivity to androgen itself. Estrogen also increases hippocampal adult neurogenesis in young and nulliparous female rats but not after middle age. On the other hand, estrogen does not promote hippocampal neurogenesis in male rats [62–65].

In addition to sex hormones, adrenal steroids have been shown to negatively regulate neurogenesis in the SGZ [66]. Furthermore, corticosteroids have been shown to cause an age-related decline in neurogenesis in the SGZ. It has also been reported that decreasing corticosteroid levels induced by removing the adrenal gland promotes neurogenesis in the SGZ of aged mice SGZ [67].

Cerebrospinal fluid, which is primarily produced by the choroid plexus and contains a number of signaling molecules, also changes with age. NSCs in the V-SVZ are in contact with cerebrospinal fluid, and age-dependent changes in the secretome in the lateral ventricular choroid plexus (LVCP) are also important for NSC regulation. Silva-Vargas et al. conducted transcriptome and proteomic assays of the LVCP secretome in young and aged mice and found that an age-dependent reduction in BMP5 and IGF1 secretion from the LVCP caused a decrease in the proliferative capacity of NSCs [68].

### Challenges of regenerative therapy involving the activation of adult NSCs

**Fig. 1:**
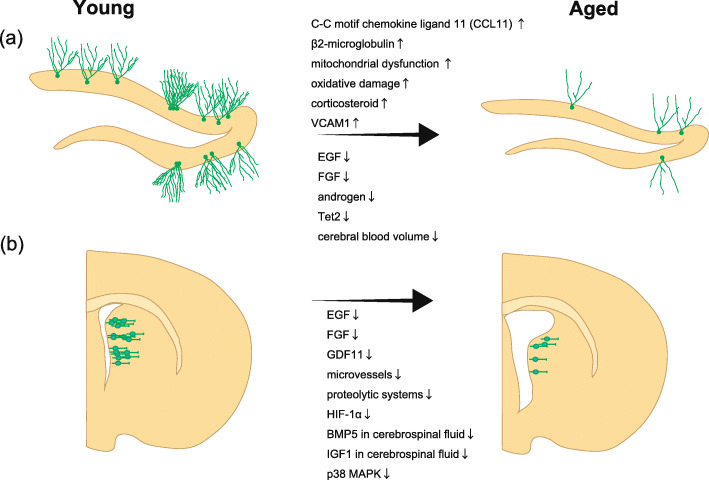
Various factors related to the decrease in adult neurogenesis with aging. Representative factors that decrease adult neurogenesis with age in the SGZ (**a**) and V-SVZ (**b**)

Adult neurogenesis is reduced due to decreases in the activity and number of NSCs caused by various factors as described above (Fig. 1). However, adult neurogenesis in the V-SVZ and DG is essential, albeit slightly, for normal brain function, such as odor discrimination, sexual behavior, and the formation of new short-term memories.

The activation of dormant NSCs to promote neurogenesis should be an effective regenerative medicine strategy for neural loss due to cerebrovascular disorders, traumatic brain injury, and neurodegenerative diseases. The activation of adult NSCs transiently increases the number of new neurons but ultimately leads to the depletion of NSCs, indicating that the supply of new neurons is limited [69, 70]. This mechanism has also been proposed in epilepsy models [71]. In epileptic seizures, abnormal firing stimulates NSCs, temporarily increasing the division of NSCs and promoting neurogenesis. However, as seizures reoccur, NSCs are depleted, eventually leading to neuronal depletion. It is not clear whether the depletion of residual NSCs induced by the activation of NSCs is harmful over a long period of time after treatment and whether long-term neurogenesis is promoted. We have found that a decrease in p38 MAPK expression in the V-SVZ, one of the areas of neurogenesis, is responsible for the age-dependent decrease in the self-proliferation of TAPs and that maintaining its expression can promote long-term adult neurogenesis without depleting NSCs [38]. Therefore, the amplification and mobilization of TAPs could be a better approach for regenerative brain repair.

## Conclusion

Since the existence of adult NSCs and adult neurogenesis was confirmed, studies on adult neurogenesis have been intensively conducted with the expectation of applying NSCs and neurogenesis for regenerative medicine.

Although the mobilization of endogenous NSCs has been studied as one of regenerative approaches to restore lost brain function in cerebrovascular diseases, traumatic brain injuries, neurodegenerative diseases, etc., there are still many issues to be solved, such as the depletion of NSCs and the directed migration of new neurons. From a fundamental point of view, identifying the regulatory mechanisms of adult neurogenesis and its age-related decline will undoubtedly lead to future regenerative medicine strategies.

## Abbreviations

aNSC: Activated neural stem cells
CCL11: C-C motif chemokine ligand 11
DG: Dentate gyrus
HIF: Hypoxia-inducible transcriptional factor
NSCs: Neuronal stem cells
qNSCs: Quiescent neural stem cells
RMS: Rostral migratory stream
SGZ: Subgranular zone
TAPs: Transit amplifying progenitor cells
Tet2: Ten-eleven translocation methylcytosine dioxygenase 2
VCAM1: Vascular cell adhesion molecule 1
V-SVZ: Ventricular-subventricular zone
VZ: Ventricular zone
